# Intracranial Melanotic Schwannomas: Rare and Distinctive Tumors to Know Due to their Risk of Recurrence and Metastases

**DOI:** 10.5334/jbsr.1359

**Published:** 2018-01-31

**Authors:** Julien Collart, Manon Vandeponseele, Pierre Bosschaert

**Affiliations:** 1 Clinique Saint-Pierre Ottignies, BE

**Keywords:** Melanotic, Schwannoma, Intracranial, Trigeminal, Gasserian

## Abstract

Melanotic schwannomas are rare nerve sheath tumors that differentiate themselves from classical schwannomas by harboring melanin-producing cells. Intracranial localizations are uncommon, especially on the Gasserian ganglion. We performed a literature review, presenting imaging characteristics, differential diagnosis, and immunohistochemical features for identification. These tumors carry the risk of recurrence and metastases. The prognosis is uncertain. We discuss recommendations for diagnosis and treatment.

## Introduction

First identified by WG Millar in 1932, melanotic schwannomas (MSs) are rare peripheral nerve sheath tumors commonly found in the thoracic paraspinal region. Extraneural locations such as the skin, bones, and viscera have also been reported. Intracranial topography is extremely rare, with 18 cases out of 105 MSs described in the literature, according to Spina et al. [1]. Only six cases involved the trigeminal nerve. These lesions distinguish themselves from classical schwannomas by the presence of cells capable of melanogenesis with intracytoplasmic pigmented granules. Therefore, the main differential diagnosis is the malignant melanoma with which they share common embryologic origin from the neural crest. MSs may have an unfavorable clinical course. Many cases of recurrence and metastases have been reported. The final preoperative diagnosis is difficult due to subtle and inconsistent imaging characteristics. We review these characteristics, offer differential diagnosis, and refer to the most recent data from literature for adequate diagnosis and treatment strategies.

## Case Report

**Figure 1:**
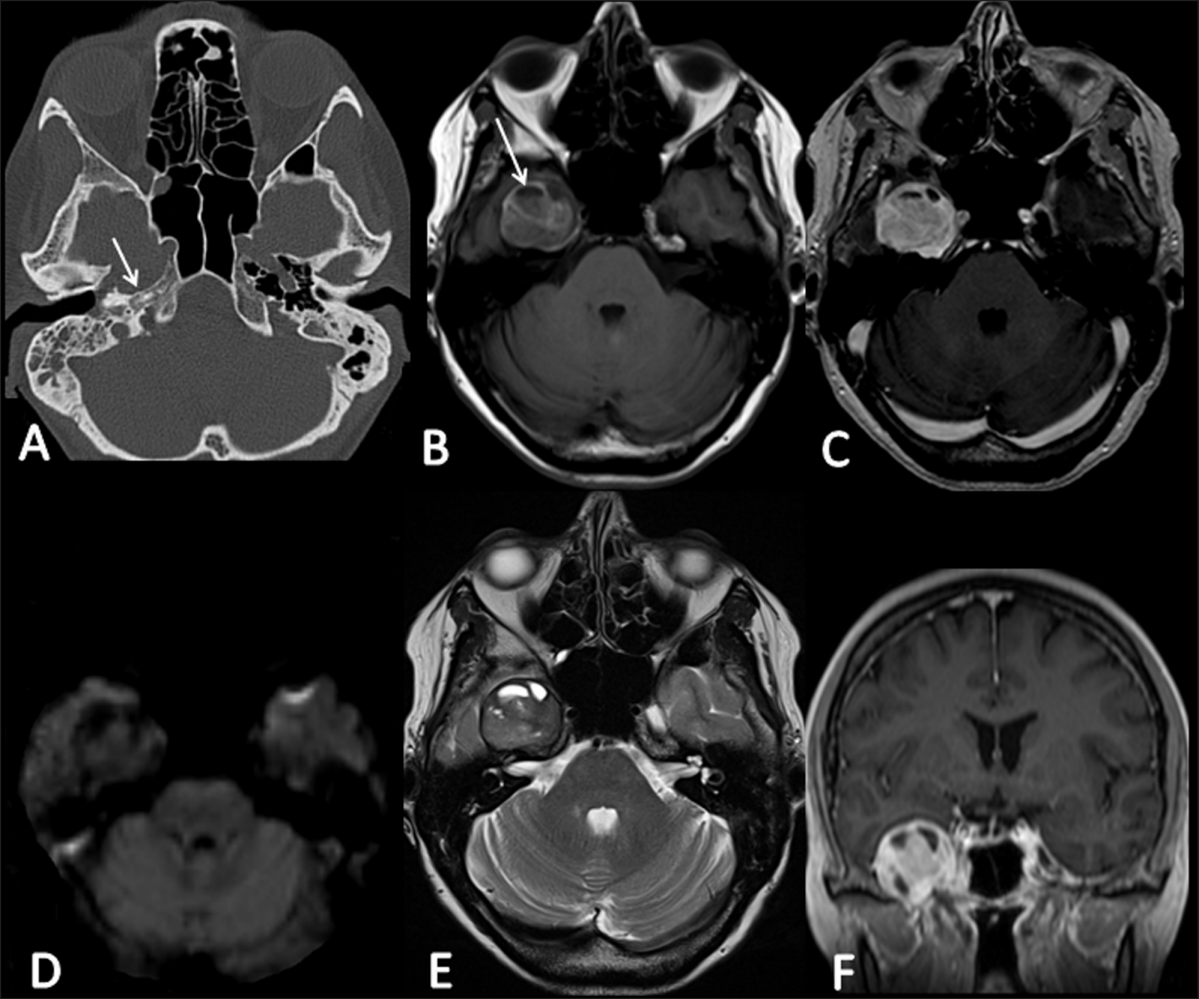
**A)** CT demonstrates a well-defined petrous lacunar image (arrow). **B)** T1-weighted image shows the hyperintense round lesion centered on the right Meckel cavum, relying on the intrapetrous carotid artery, and generating a mass effect on the temporal lobe (arrow). **D)** DWI is negative. **E)** T2-weighted image shows the presence of cystic areas. **C)** and **F)** Axial and coronal T1-weighted images show a slightly heterogeneous enhancement after contrast administration.

**Figure 2:**
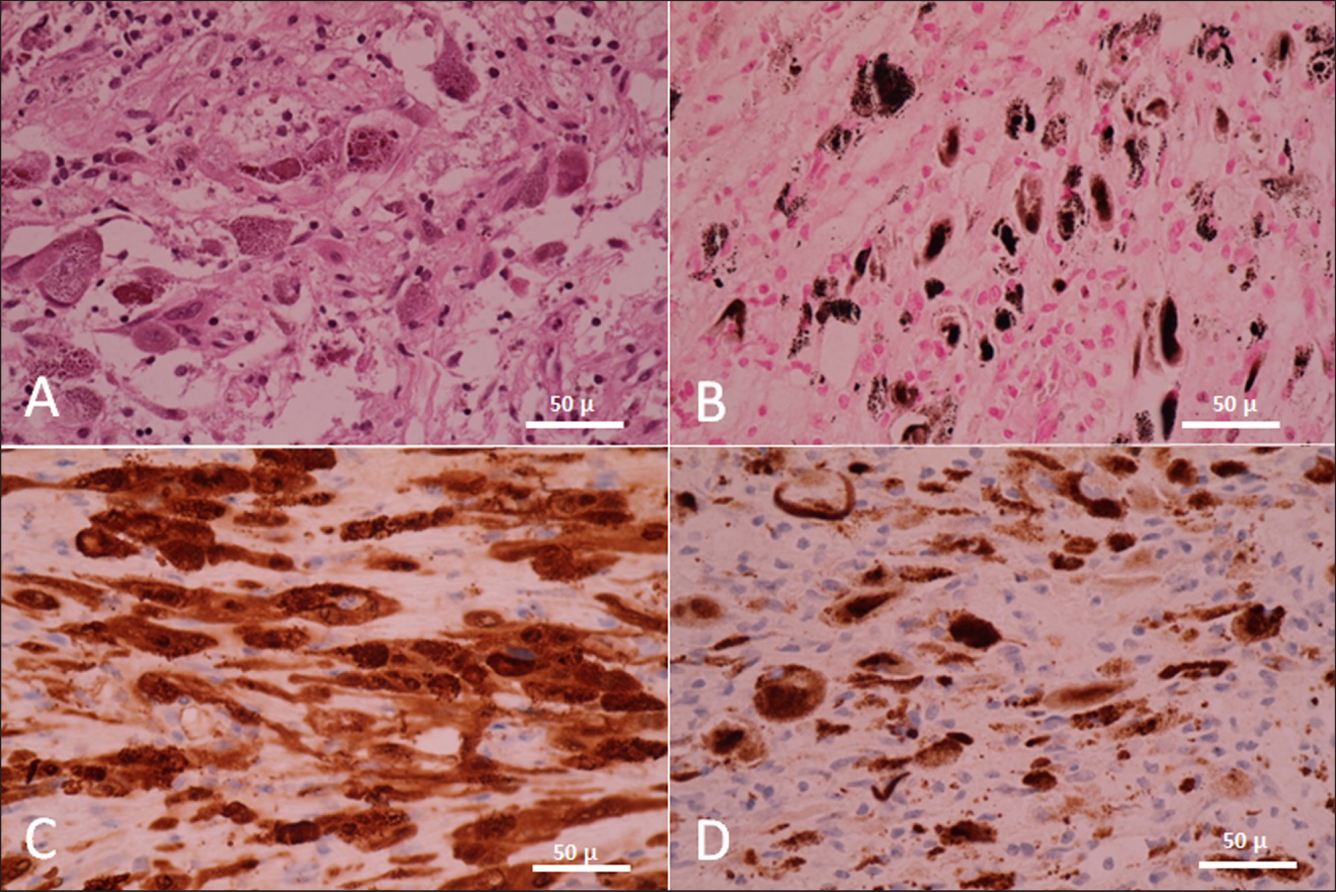
Microscopic pictures of tumor cells (×40) reveal their dual Schwann and melanocytic phenotype. **A)** Hematoxylin-eosin staining shows plump pigmented tumorous Schwann cells. No psammomatous bodies are observed. **B)** Fontana staining shows the presence of melanin in tumorous Schwann cells. **C)** S-100 protein and **D)** HMB-45 confirm the diagnosis.

**Figure 3:**
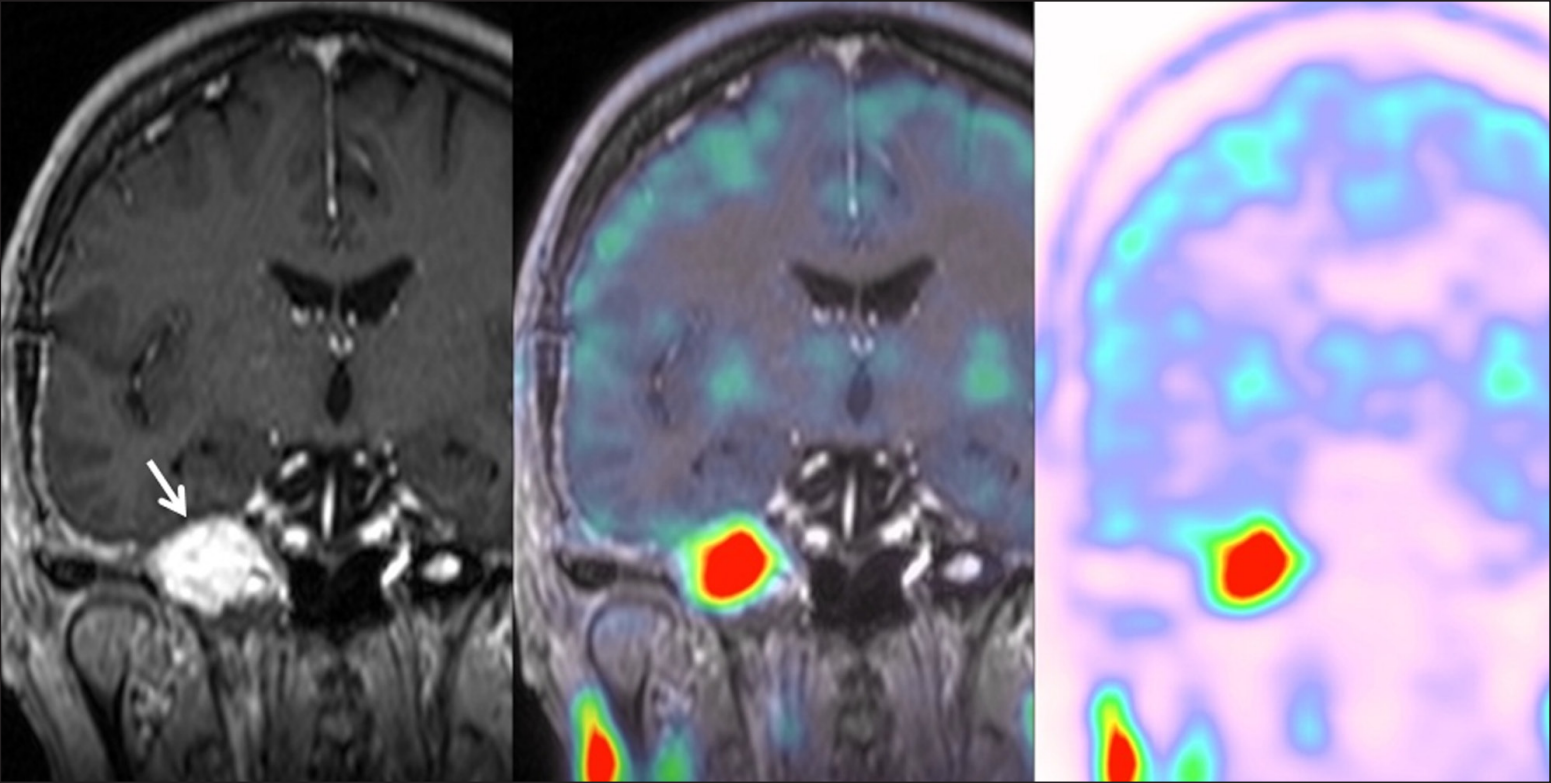
MET-PET/MRI shows the recurrence with a hypermetabolic activity (arrow).

A 64-year-old woman was admitted to the neurosurgery department for V2–V3 right- sided trigeminal paresthesia, which had developed six months earlier. Magnetic resonance imaging (MRI) showed a well-circumscribed, fleshy, 36 mm tumor in the Meckel cavum involving the Gasserian ganglion, following the V3 nerve into the foramen ovale, and generating a mass effect on the temporal lobe. This lesion was discreetly hyperintense on T1-weighted images and iso- to hypointense on T2-weighted images, and it showed a slightly heterogeneous enhancement after contrast administration. Small cystic areas were noted. Diffusion-weighted imaging was negative. An abnormality was already seen on a computed tomography (CT) performed two years previously in the context of otitis, without any alarm in relation to the benign presentation, a well-defined petrous lacunar image, and the fortuitousness of the discovery (Figure 1). Diagnosis of schwannoma was proposed, without specification. The patient underwent a macroscopically complete surgical resection by an exclusive extradural subtemporal approach with excellent symptomatic recovery. The postoperative CT demonstrated no complications. Immunohistochemical examination finally specified the diagnosis of MS, a very rare pigmented tumor (Figure 2). The clinical course was good. However, a recurrence was detected on the MRI performed at three months. 11C-methionine positron emission tomography (MET-PET) confirmed suspicions (Figure 3), and a new surgical intervention was planned followed by adjuvant radiotherapy.

## Discussion

MSs are rare nerve sheath tumors that differentiate themselves from classical schwannomas by harboring melanin-producing cells. Intracranial localization is uncommon, especially on the Gasserian ganglion. In the literature, only six cases involved the trigeminal nerve. Twenty percent of patients have multiple locations. Peak incidence is between the fourth and fifth decade of life, without any sex predominance [2]. MSs can be sporadic or part of neurofibromatosis type II or Carney complex, an autosomal-dominant disease that includes skin pigmentary abnormalities, myxomas, and endocrine tumors (Cushing syndrome, precocious puberty, and acromegaly). Carney described this complex as being associated with a distinct pattern of MSs characterized by the presence of psammomatous bodies at histologic examination. However, two cases of nonpsammomatous MSs associated with Carney complex were reported. Thus, the lack of psammomatous bodies does not exclude Carney complex. Recently, the inactivating mutation of the tumor-suppressor gene *PRKAR1A* was found in about 50% of Carney complex cases [3,4]. In our case, no psammomatous bodies were found, and there was no other endocrine, cutaneous, or conjunctive detected abnormality, or family history.

On CT, MSs generally appear isodense or slightly hyperdense and may contain calcifications. Bone impressions can be observed. On MRI, these lesions usually appear hyperintense on T1-weighted sequences and iso- to hypointense on T2-weighted sequences because of the intracellular storage of melanin. In comparison, classical schwannomas appear hypointense on T1-weighted sequences and hyperintense on T2-weighted sequences. Contrast enhancement after gadolinium administration may be homogeneous or heterogeneous in relation to the degree of cellularity, cysts, calcifications, or hemorrhage areas. The differential diagnosis should include other melanotic lesions, such as malignant melanoma, pigmented meningioma, and medulloblastoma [5].

Most often, histologic examination shows highly cellular tumors composed of spindle-shaped and epithelioid cells with an eosinophilic-to-amphophilic cytoplasm. Lesions are well-circumscribed and may contain calcifications and hemorrhage areas. The cells stain positive for melanin (Fontana), reticulin (Gomori), and periodic acid-Schiff. Psammomatous bodies must be searched. MSs also express S-100 protein, neuron-specific enolase (NSE), vimentin, and stain variably with Melan-A, HMB-45, and other melanocytic markers. Antigen Ki-67 shows a highly variable mitotic index between 1% and 20%, with an average of 3% [6].

MSs may have a malignant clinical course even in the absence of histologic criteria of malignancy. Recurrence and metastases are observed 20 years after diagnosis. Furthermore, intracranial topography presents a higher risk [7,8,9]. The main site of metastasis is the lung.

Surgical resection is the treatment of choice. In the literature, out of 16 patients with intracranial MS who have undergone surgical treatment, total resection was completed in eight patients. In this resection group, recurrence was achieved in 25% compared with 50% in the subtotal resection group. Adjuvant radiotherapy remains controversial. Watson et al. [1] suggested that, without histologic signs of malignancy, adjuvant treatment should not be performed and only strict radiologic and clinical follow-up is required. In our case, the tumor only presented a focally high Ki-67 index of 18%. This was falsely reassuring. Because of the potential malignant behavior of these tumors, other authors emphasize the role of adjuvant radiotherapy in case of subtotal resection. Our patient underwent a macroscopically complete resection. Vallat-Decouvelaere and Zhang [1,6] observed a lower rate of recurrence and metastases in patients with MS treated with adjuvant radiotherapy. According to these authors, radiotherapy should be considered in case of histologic criteria of malignancy, incomplete surgical resection, tumor recurrence, or metastases. Despite encouraging data, no clinical series have been published to date on the effectiveness of radiotherapy, and no treatment protocol is available. This is the reason that our multidisciplinary team proposed, obviously wrongly, adjuvant radiotherapy only at the time of recurrence.

In conclusion, intracranial MSs are very rare and can mimic classical schwannomas. Although the definite diagnosis is often based on immunohistochemical analysis, MRI can play an important role in the diagnostic approach if radiologists know of this entity’s existence. Recurrence and metastases are possible. Total resection is clearly recommended, and postoperative adjuvant radiotherapy should be considered in the histologic confirmation of the diagnosis, even if the imaging is reassuring and the resection macroscopically complete, the prognosis being uncertain. Long-term follow-up is required.
